# How science and policy came together and made a global impact: The EU common list of COVID-19 antigen tests

**DOI:** 10.12688/openreseurope.18267.1

**Published:** 2024-09-09

**Authors:** Barbara Raffael, Mauro Petrillo, Gabriele Leoni, Tobias Wiesenthal, Yoline Kuipers, Maddalena Querci

**Affiliations:** 1 European Commission Joint Research Centre (JRC), Ispra, Italy; 2 Seidor Italy S.r.l., Milan, 20129, Italy; 3 European Commission Joint Research Centre (JRC), Geel, Belgium; 4 European Commission, Directorate-General Health and Food Safety (SANTE), Luxembourg, Luxembourg

**Keywords:** COVID-19, policy support, science for policy

## Abstract

Science can play a pivotal role in providing support to public health policy making processes, and to ensure effective and efficient implementation of policies.

This work illustrates how science was integrated into policymaking during the peak of the COVID-19 pandemic, in relation to countries’ COVID-19 antigen testing strategies as well as the implementation of Regulation (EU) 2021/953 on the EU Digital COVID Certificate. The lessons learnt during this process as well as the critical steps taken, and concrete recommendations, have been identified and capitalised and turned into a list of science-based advice. The availability of an already established mechanism that can be quickly adapted in case of need, is likely to be highly beneficial in case of a future public health emergency.

## Plain language summary

This open letter illustrates how science was integrated into policymaking during the peak of the COVID-19 pandemic, in relation to countries’ COVID-19 antigen testing strategies as well as the implementation of Regulation (EU) 2021/953 on the EU Digital COVID Certificate. The lessons learnt during this process as well as the critical steps taken, and concrete recommendations, have been identified and capitalised and turned into a list of science-based advice.

We consider that the availability of an already established mechanism that can be quickly adapted in case of need, is likely to be highly beneficial in case of a future public health emergency.

## Introduction

Since the outbreak of COVID-19 pandemic, testing strategies were a critical component in the EU’s efforts to prevent, contain and mitigate the impact of the disease. Identifying infected individuals and preventing further virus transmission was essential to rapidly mitigate the pandemic, and for its subsequent control, allowing citizens to restart travelling and re-joining social activities and work-related tasks. The European Commission (EC) adopted various policy documents and legal acts to support Member States (MS) in the implementation of their COVID-19 testing strategies ^1^. The EU Digital COVID Certificate (EUDCC) was introduced on July 1, 2021 ^2^, serving as the digital proof that, amongst others, a person had received a negative COVID-19 test result.

**Figure 1.**
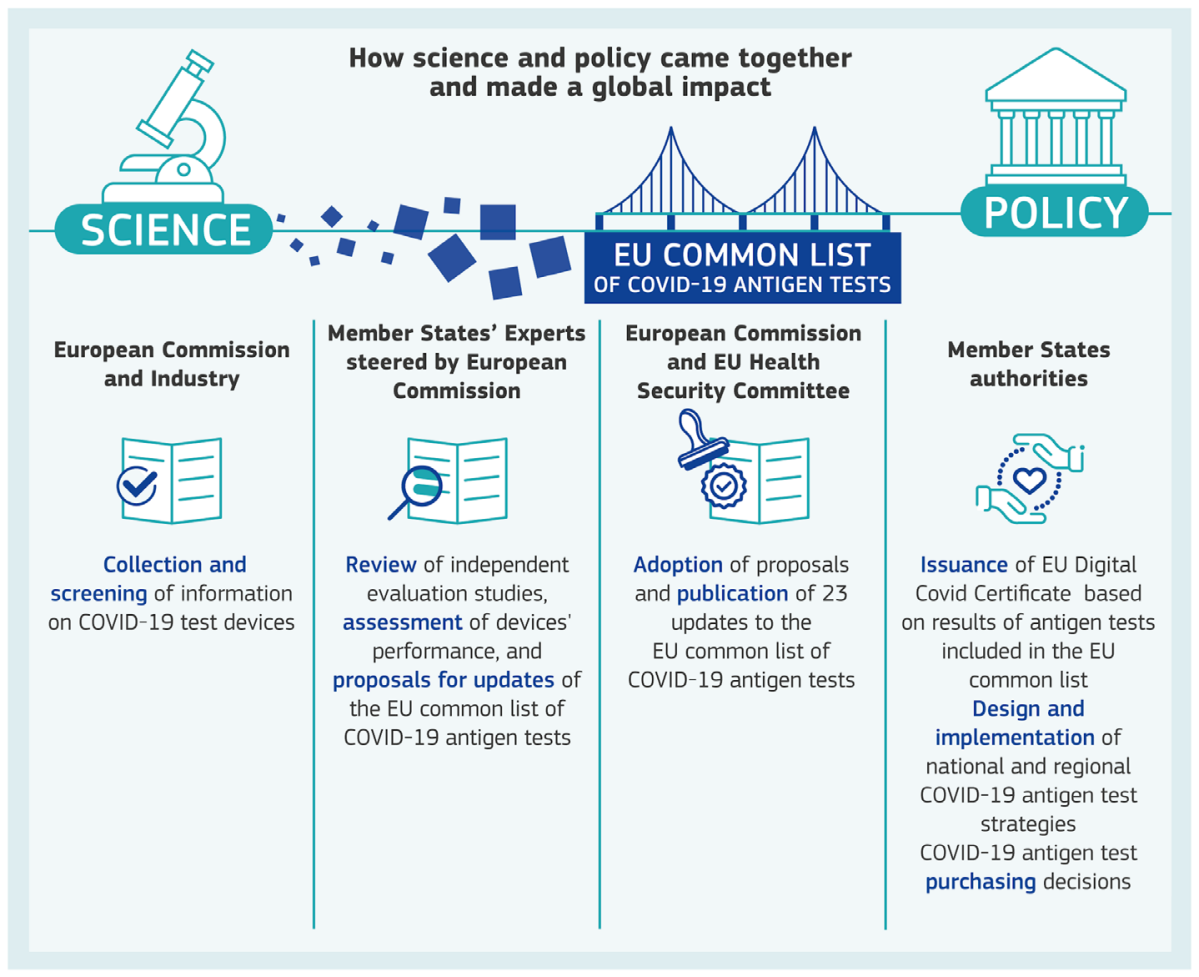
Science and policy together. Visual of how science and policy came together and made a global impact for ensuring safe and reliable COVID-19 antigen testing.

To support EU level public health decision-making on COVID-19 antigen testing, a scientific process was put in place, consisting of three connected parts, for i) information rationalisation, ii) integration of science into policy, and iii) benefits for citizens via the EUDCC. A graphical overview of the whole process for ensuring safe and reliable COVID-19 antigen testing is presented in Figure 1.

## Policy outcomes and implications

### Information rationalisation

At the start of the pandemic, evidence on efficiency and reliability of COVID-19 antigen testing policies as well as available antigen devices was scarce. Later during the pandemic, the situation turned around and available information became extensive but scattered. There was therefore a need to create a platform to make information and data provided by the research community, as well as manufacturers, available in one place.

The COVID-19 in Vitro Diagnostic Devices and Test Methods Database (DB) was developed ^3^ to meet this need. It stands as one of the most comprehensive inventories of COVID-19 testing devices and methods globally. It makes relevant information on the performance of in vitro diagnostic medical devices and laboratory-developed methods for COVID-19 publicly available and up to date. With more than 2 600 testing devices and methods stored, it has been accessed by visitors from more than 168 countries ^4^.

### Integration of science into policy

Among the testing possibilities, rapid antigen tests (RATs), despite being less accurate than PCR tests, are much faster, cheaper, and easy to use. Consequently, the role played by COVID-19 RATs became significant, and the number of devices available on the EU market increased exponentially during the pandemic.

In September 2020, the EU Health Security Committee (HSC) began gathering data on RATs performance from evaluation studies conducted within MS.

Following this, the Council of the European Union issued a Council Recommendation ^5^ in January 2021. This recommendation called upon EU MS to agree on and maintain a common list of COVID-19 antigen tests, and to mutually recognise the result of those tests included. As a result, the first EU common list of COVID-19 antigen tests was published in February 2021.

Initially, device inclusion in the list was based on market availability and manufacturer’s claims. However, RATs efficacy was vital for the effectiveness of the EUDCC implementation, needing an efficacy verification system, through a transparent evaluation procedure with well-defined scientific criteria. For this, in May 2021, the HSC set up a specialised Technical Working Group on COVID-19 diagnostic tests (TWG). The purpose of the TWG was to evaluate submissions from countries and manufacturers for devices to be included to the EU common list, based on agreed criteria. Furthermore, the TWG, along with the Commission and the European Centre for Disease Prevention and Control, was tasked with drafting a technical guidance for the independent validation of the devices.

A dedicated system (based on EUSurvey, CIRCABC and the DB) was developed and used by manufacturers to upload applications, and by the TWG to collect relevant information and data to be reviewed for their devices assessment for possible inclusion in the EU common list. The system ensured confidentiality and expert anonymity while swiftly managing sensitive information. This workflow facilitated the integration of science into policy. It allowed for the responsibility of EU common list modifications to remain in MS’ hands, through the HSC, while ensuring that decisions were based on scientifically solid evaluations.

Since its first publication, the EU common list has undergone 23 revisions, taking into account the results of new validation studies, the introduction of new devices to the market, the epidemiological developments, and the emergence of new SARS-CoV-2 variants. The latest version of the list can be found on the TWG COVID-19 diagnostic tests webpage ^6^.

As of 1 July 2021, under the EUDCC Regulation (EU) 2021/953, all devices enclosed in the EU common list and carried out by health professionals or qualified testing personnel could be used by MS to issue EUDCC test certificates, and (as of 22 February 2022) EUDCC recovery certificate.

### Benefit for citizens via the EUDCC

The DB was updated and adapted to include the evolving EU common list. The list was automatically extracted from the database on daily basis. The extracted information, converted into a language-independent data format file (JSON), was and still is accessible at a static URL ^7^.

This served as the foundation for the information displayed on the EUDCC test and recovery certificates that were based on antigen testing. The EUDCC facilitated safe travel for citizens across the European Union and allowed to coordinate the lifting of restrictions when possible.

**Figure 2.**
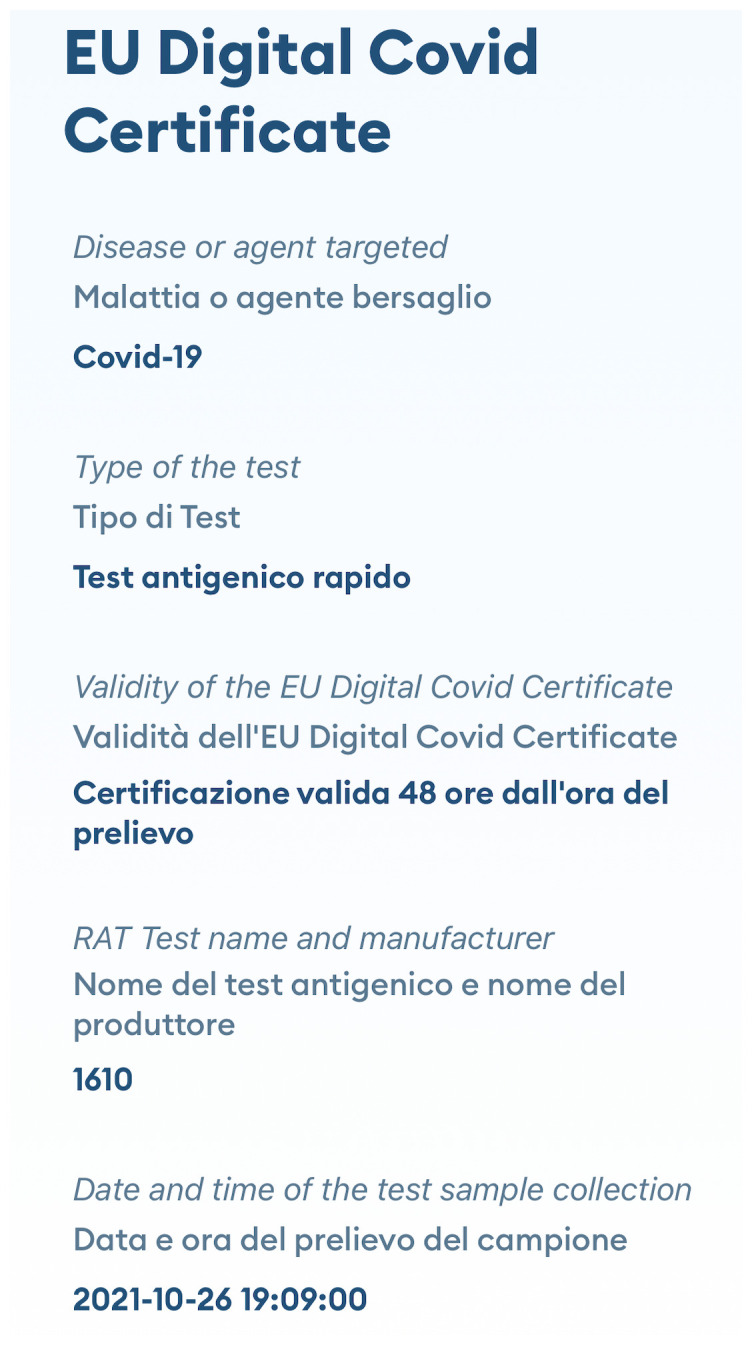
Example of digital EUDCC. The figure is the mobile screenshot of an EUDCC issued in Italy after testing, in which “RAT Test name and manufacturer” are expressed as the numerical code (1610) assigned to the antigen tests in the COVID-19 in Vitro Diagnostic Devices and Test Methods Database.

The EUDCC has also been a success worldwide: it has set a global standard for international travel and has been the only system in operation at international level. An example of digital EUDCC is presented in Figure 2.

## Actionable recommendations

The capitalization of lessons learned in the context of the EU common list and its related evaluation process by experts can be precious for future needs, as they can facilitate the development of an workflow in case of future public health emergencies.

The work that was carried out by the TWG came with a heavy workload that required proper and efficient management. In total, the TWG reviewed over 1 100 manufacturer applications, declining over time without reaching a plateau. To support their work, the Commission screened applications for compliance before transmitting them to TWG experts, significantly reducing the TWG workload by approximately 40%. For future needs, it is recommended to foresee a screening process with dedicated personnel.

EC platforms were used for information exchange and document management. When the amount of documents to be included in the manufacturer’s applications increased, due to evaluation criteria becoming stricter, the limits of the platforms capacity became a limiting factor. Future systems should be more flexible or anticipate increased documentation load.

Dedicated mailboxes were established for communication with manufacturers and TWG: the volume of non-compliant applications and support requests reached an overwhelming amount of over 18 000 emails. Thus, adequate resources for help-desk staff are recommended for future needs.

The TWG conducted independent evaluations of applications, with plenary meetings both for reaching consensus and for revising requirements. While written procedures were employed when a meeting could not be arranged, meetings demonstrated to be essential for discussing device performance and revising guidelines and criteria. Adequate resources for the organisation of regular meetings are recommended for future planning.

The initial version of the common list featured 26 devices, reaching 279 devices by the end of the exercise. The dynamic list required a comprehensive management approach, leading to the adoption of a "master file" for discussions and assessments. Future planning should anticipate such solution and allocate necessary resources accordingly.

Challenges, solutions, and recommendations are summarised in Table 1.

**Table 1.** Challenges, solutions and recommendations.

Challenge	Adopted solution	Recommendation
High and irregular workload for experts	Initial screening of data	Foresee skilled support staff
High volume of confidential data	Platforms for information exchange and for document management and storage	Foresee a flexible and scalable system since the beginning
Need for constant exchange of information with manufacturers	E-mail help desk	Foresee flexible staff for secretarial support
Need to discuss data and criteria	Written procedure and organisation of meetings	Foresee organization of regular meetings
Enormous amount of data processed, re-processed and adjusted, and relative discussions, outcomes and assessments	Creation of a master-file	Allocation of resources for file management

## Conclusions/Discussion

The crisis prompted the development of an action protocol that had a direct impact on the health and well-being of EU citizens. It facilitated secure information exchange between manufacturers and experts, with swift adaptability and robust security measures. Within the EU, it allowed to agree on a list of antigen tests that were meeting common criteria. This supported countries in developing and implementing COVID-19 testing strategies as well as issuing the relevant EUDCC test and recovery certificates. In addition, the EU common list provided support to MS procurement procedures and national insurance and reimbursement schemes. At the global level, it also facilitated travelling through the EUDCC, and indirectly provided quality standards to be used as a global reference. In total, 51 countries across four continents have benefited from the EUDCC system, lately adopted (including its links to the DB) as the first building block of the WHO Global Digital Health Certification Network that will develop digital products “ *to deliver better health for all*” ^8^.

A unified evaluation framework by Member State experts reduced duplication of efforts and ensured uniform decisions, benefiting all. Furthermore, industry had to follow guidelines based on the scientific criteria set by the TWG.

The coordinated EU response minimized duplication and conflicting standards among Member States, enabling faster citizen-centric actions and knowledge sharing. To ensure a swift and effective response to future emergencies, key proposed actions include the immediate nomination of a Team of Experts, the establishment of an independent management team, the development of working procedures and performance criteria, and the creation of detailed guidance and of a solid information exchange system.

Lessons learnt are crucial for future preparedness.

The universality of the scientific method transcends political boundaries, ensuring its applicability across diverse contexts. As evidenced by the adoption of the EUDCC not only in Europe but also in nations worldwide, our reliance on the scientific method underscores its efficacy in informing evidence-based policy decisions globally.

## Disclaimer

The views expressed in this article are those of the author(s). Publication in Open Research Europe does not imply endorsement of the European Commission.

## List of abbreviations

**CIRCABC**: Communication and Information Resource Centre for Administrations, Businesses and Citizens.

**DB**: COVID-19 in Vitro Diagnostic Devices and Test Methods Database

**EU**: European Union

**EUDCC**: EU Digital COVID Certificate

**EUSurvey**: European Union Survey management system

**HSC**: Health Security Committee

**MS**: Member States

**RATs**: Rapid antigen tests

**TWG**: Technical Working Group on COVID-19 diagnostic tests

## Data Availability

No data are associated with this article. BR, MP drafted the manuscript. BR, MP, GL, MQ provided technical comments to improve the manuscript. BR, MP, GL, MQ reviewed & edited. All authors read and approved the final manuscript.
