# Supplementary material for: How science and policy came together and made a global impact: The EU common list of COVID-19 antigen tests
Source: Open Res Eur. 2024 Dec 24;4:198. Originally published 2024 Sep 9. [Version 2] doi: 10.12688/openreseurope.18267.2 (PMC11809469; doi:10.12688/openreseurope.18267.2)
Supplement: Peer review history

# Reviewer report: Reviewer response for version 2

**DOI:** 10.21956/openreseurope.20756.r49507

**Published:** 2025-01-23

**Authors:** Carles Muntaner, Virginia Gunn, Yining Dai

**Affiliations:** 1 UPFD Barcelona School of Mnagement and JH-UPF Public Policy Center (JHU-UPF PPC), Universitat Pompeu Fabra, Barcelona, Catalonia, Spain; 2 University of Toronto, Toronto, Ontario, Canada; 3 Nursing, Cape Breton University, Sydney, Nova Scotia, Canada; 4 Computing and Communication, Toronto Metropolitan University Yeates School of Graduate Studies, Toronto, Ontario, Canada

The revised manuscript addresses reviewer comments to enhance its clarity and relevance for public health decision-making, particularly in future pandemic preparedness. It focuses on the EU’s COVID-19 antigen testing strategies and evidence-to-policy translation, emphasizing the utility of rapid antigen tests (RATs) for detecting infectiousness.

 Testing strategies were pivotal in mitigating COVID-19's spread, enabling individuals to resume social and economic activities. The European Commission (EC) introduced policies like the EU Digital COVID Certificate (EUDCC) to standardize and implement testing across Member States (MS). A scientific workflow comprising evidence identification, policymaking, and implementation was established, supported by a comprehensive database of in vitro diagnostic devices and methods.

 RATs, though less precise than PCR tests, provided faster, cheaper, and widely deployable testing. To evaluate and maintain a common list of RATs, the EU established a Technical Working Group (TWG), which assessed over 1,100 manufacturer applications using criteria like sensitivity, specificity, and validation by at least one MS. The list underwent 23 updates, reflecting new scientific data and market developments.

 The EUDCC, enabled by this process, became a global standard for travel and public health coordination, adopted by 51 countries and forming a foundation for the WHO Global Digital Health Certification Network. Lessons learned include the need for scalable systems, expert support, and efficient communication. These findings are vital for future preparedness, emphasizing the importance of evidence-based, globally coordinated public health policies to address emerging crises.

 Review: Where is the Global Impact of the EU?

 By a narrow focus on the EU common list of Antigen tests  the authors ignore the critiques of the public health responses to the COVID-19 pandemic, often dealing with a lack of coordination at the EU level. For example, differences in preparedness, political decision-making, and public health infrastructure. Here are some insights drawn from the research:

Lack of Consistent Preparedness: Many countries faced challenges in testing, contact tracing, and communication with the public, leading to a fragmented response. An analysis of 28 countries highlighted resilience in some areas but underscored gaps in coordination and timely implementation of public health measures ( Haldane et al. Ref 1).

Delayed Responses: Delays in lockdowns, inconsistent enforcement of social distancing measures, and inadequate initial responses to outbreaks were frequently criticized. For example, countries like Italy and Spain faced criticism for failing to act swiftly, amplifying the impact of early outbreaks ( Carta et al. Ref 2).

Communication Failures: Effective public health communication was inconsistent across EU members. Research into the use of social media platforms by public health authorities showed inequities in reaching diverse populations effectively, which hampered public trust and adherence to guidelines ( Raamkumar et al. Ref 3).

Equity and Access Issues: vaccination rollouts and access to healthcare resources revealed significant inequalities. In some countries, deprived and discriminated populations faced systemic barriers to testing and vaccination ( Edwards & Ott Ref 4).

Overreliance on Centralized Approaches: Critiques of centralized strategies in regions such as the EU pointed to inefficiencies and failures in coordinating cross-border health measures. A review highlighted how health governance was strained under the demands of the pandemic ( Greer et al. Ref 5).

Failure to learn from lessons from comparative analysis: Countries with robust prior investments in public health infrastructure, such as South Korea, managed testing and contact tracing more effectively during the first wave. This highlighted how pre-existing systems and rapid mobilization were crucial in mitigating the pandemic's effects ( Assefa et al. Ref 6).

 Global inequities: the pandemic is a case study to illustrate how misplaced priorities—emphasizing market values over human ones—left societies vulnerable. The EU did not prioritize equity, resilience, and sustainability, highlighting the lack of interconnectedness of health, economics, and global cooperation with low-income countries lacking acces to vaccines (Carney, M Value(s): Building a Better World for All Penguin Canada )

 These critiques underscore the need for enhanced global preparedness, equitable healthcare access, and improved communication strategies for managing future public health crises.

Where applicable, are recommendations and next steps explained clearly for others to follow? (Please consider whether others in the research community would be able to implement guidelines or recommendations and/or constructively engage in the debate)

Yes

Does the article adequately reference differing views and opinions?

No

Are all factual statements correct, and are statements and arguments made adequately supported by citations?

Partly

Is the rationale for the Open Letter provided in sufficient detail? (Please consider whether existing challenges in the field are outlined clearly and whether the purpose of the letter is explained)

Partly

Is the Open Letter written in accessible language? (Please consider whether all subject-specific terms, concepts and abbreviations are explained)

No

Reviewer Expertise:

Social inequalities in health, health equity

We confirm that we have read this submission and believe that we have an appropriate level of expertise to confirm that it is of an acceptable scientific standard, however we have significant reservations, as outlined above.

## References

[1] : Health systems resilience in managing the COVID-19 pandemic: lessons from 28 countries. *Nat Med* .2021;27(6) : 10.1038/s41591-021-01381-y 964-980 10.1038/s41591-021-01381-y 34002090

[2] : Comparing the responses of countries and National Health Systems to the COVID-19 pandemic: a critical analysis with a case-report series. *Eur Rev Med Pharmacol Sci* .2023;27(16) : 10.26355/eurrev_202308_33442 7868-7880 10.26355/eurrev_202308_33442 37667964

[3] : Measuring the Outreach Efforts of Public Health Authorities and the Public Response on Facebook During the COVID-19 Pandemic in Early 2020: Cross-Country Comparison. *J Med Internet Res* .2020;22(5) : 10.2196/19334 e19334 10.2196/19334 32401219 PMC7238862

[4] : Governments’ Responses to the COVID-19 Pandemic. *International Journal of Public Administration* .2021;44(11-12) : 10.1080/01900692.2021.1936964 879-884 10.1080/01900692.2021.1936964

[5] : Do coastal salt mudflats (sabkhas) contribute to the blue carbon sequestration?. *Biogeochemistry* .2025;168(1) : 10.1007/s10533-024-01204-5 15 10.1007/s10533-024-01204-5 39829715 PMC11735541

[6] : Analysis of the COVID-19 pandemic: lessons towards a more effective response to public health emergencies. *Global Health* .2022;18(1) : 10.1186/s12992-022-00805-9 10 10.1186/s12992-022-00805-9 35120537 PMC8815718

# Response

**Published:** 2025-01-24

**Authors:** Barbara RAFFAEL

Dear Reviewers,

 Thank you for your detailed comments on our open letter. We appreciate the time and effort you have taken to provide this feedback. We would like to clarify that the primary focus of our open letter is the analysis of the path towards the formulation of the EU common list of COVID-19 antigen tests.

 Our aim is to highlight the importance of the scientific input in the implementation of policy decisions. Our document intends to provide only scientific advice and practical recommendations that may help in addressing future health crises, in the field of testing. It is not meant to address broader critiques of public health responses, political decision-making, or the management of public health and public health infrastructure at either the EU or Member States level.

 Consequently, we believe that your concerns fall outside the scope of our current study. Thus, our intention is to maintain a focused analysis on the procedural aspects that led to the EU common list of antigen tests, without incorporating political considerations or broader public health issues.

 We hope this clarifies the scope and intent of our open letter. Thank you once again for your feedback.

 Sincerely,

 Barbara Raffael, on behalf of all the authors

# Reviewer report: Reviewer response for version 2

**DOI:** 10.21956/openreseurope.20756.r49122

**Published:** 2025-01-08

**Authors:** Benjamin Vallejo

**Affiliations:** 1 University of the Philippines Diliman, Quezon City, Philippines

Thank you. The references to the information on how accurate RAT tests in the EU will be useful for public health authorities.

Where applicable, are recommendations and next steps explained clearly for others to follow? (Please consider whether others in the research community would be able to implement guidelines or recommendations and/or constructively engage in the debate)

Yes

Does the article adequately reference differing views and opinions?

Yes

Are all factual statements correct, and are statements and arguments made adequately supported by citations?

Partly

Is the rationale for the Open Letter provided in sufficient detail? (Please consider whether existing challenges in the field are outlined clearly and whether the purpose of the letter is explained)

Yes

Is the Open Letter written in accessible language? (Please consider whether all subject-specific terms, concepts and abbreviations are explained)

Yes

Reviewer Expertise:

Science advice and policy, marine environmental science, science, technology and society

I confirm that I have read this submission and believe that I have an appropriate level of expertise to confirm that it is of an acceptable scientific standard.

# Reviewer report: Reviewer response for version 2

**DOI:** 10.21956/openreseurope.20756.r49121

**Published:** 2024-12-27

**Authors:** Iain Buchan

**Affiliations:** 1 University of Liverpool, Liverpool, UK

Thank you for addressing all of the points raised in my review of Version 1. This version of the paper reads well and concisely conveys an important message about trans-national preparedness for pandemic testing.

Where applicable, are recommendations and next steps explained clearly for others to follow? (Please consider whether others in the research community would be able to implement guidelines or recommendations and/or constructively engage in the debate)

Partly

Does the article adequately reference differing views and opinions?

Partly

Are all factual statements correct, and are statements and arguments made adequately supported by citations?

Partly

Is the rationale for the Open Letter provided in sufficient detail? (Please consider whether existing challenges in the field are outlined clearly and whether the purpose of the letter is explained)

Partly

Is the Open Letter written in accessible language? (Please consider whether all subject-specific terms, concepts and abbreviations are explained)

Partly

Reviewer Expertise:

Public Health, Data Science, COVID-19/pandemic testing

I confirm that I have read this submission and believe that I have an appropriate level of expertise to confirm that it is of an acceptable scientific standard.

# Reviewer report: Reviewer response for version 1

**DOI:** 10.21956/openreseurope.19739.r46416

**Published:** 2024-12-05

**Authors:** Benjamin Vallejo

**Affiliations:** 1 University of the Philippines Diliman, Quezon City, Philippines

Rapid antigen tests (RAT) while less accurate, proved valuable in detecting COVID-19 positive patients so that interventions can be done immediately. This proved important in determining quarantine status and whether to relax or maintain travel and mobility restrictions. I would suggest the authors to present an estimate of how accurate the RAT tests used in the EU are and the general criteria used to determine which are acceptable and which are not. That's because the EU agreed on a list of RAT tests. This information has to be presented as it is important in implementing quarantine methods just in case another global pandemic happens.

Where applicable, are recommendations and next steps explained clearly for others to follow? (Please consider whether others in the research community would be able to implement guidelines or recommendations and/or constructively engage in the debate)

Yes

Does the article adequately reference differing views and opinions?

Yes

Are all factual statements correct, and are statements and arguments made adequately supported by citations?

Partly

Is the rationale for the Open Letter provided in sufficient detail? (Please consider whether existing challenges in the field are outlined clearly and whether the purpose of the letter is explained)

Yes

Is the Open Letter written in accessible language? (Please consider whether all subject-specific terms, concepts and abbreviations are explained)

Yes

Reviewer Expertise:

Science advice and policy, marine environmental science, science, technology and society,

I confirm that I have read this submission and believe that I have an appropriate level of expertise to confirm that it is of an acceptable scientific standard, however I have significant reservations, as outlined above.

# Response

**Published:** 2024-12-12

**Authors:** Mauro PETRILLO

Thank you for your thoughtful comments on our paper. We agree that providing an estimate of the accuracy of RAT tests used in the EU and the criteria for determining their acceptability would be a valuable addition. We addressed this point in the section “Evidence-based Policymaking” by adding information on the acceptance criteria and the percentage of devices that did not meet the agreed standards, to highlight the importance of the evaluation exercise in maintaining a suitable level of accuracy for the devices used to issue the EUDCC. More detailed information on these topics can also be found in the referenced JRC Science for Policy Report (doi: 10.2760/573813).

# Reviewer report: Reviewer response for version 1

**DOI:** 10.21956/openreseurope.19739.r46414

**Published:** 2024-11-25

**Authors:** Iain Buchan

**Affiliations:** 1 University of Liverpool, Liverpool, UK

Important topic. Lessons from rapid antigen testing in COVID-19 responses that are key to future pandemic preparedness. The learning is also applicable to minimising avoidable gaps between evidence availability and policy implementation across a wide range of topics of public health importance.

 The article might better draw upon related multi-stakeholder reflections such as: https://www.thepandemicinstitute.org/collaborate/academics/community-testing-review/, where experts (mostly from the UK COVID-19 Testing Initiatives Evaluation Board) reflected on SARS-CoV-2 rapid antigen testing uses, evaluations and impacts.

 The article needs to make the purposes for testing (e.g. Ref 1, 9) clearer. Terms "effectiveness" "efficiency" "reliability" "accuracy" need a context of use and a defined utility e.g. for minimising the time taken to identify that a person is likely infectious and to inform their (or other person's) actions such as self-isolation, minimising social mixing, early return from quarantine or isolation to work...

 The three stages of "information rationalisation", "integration of science into policy" and "benefits for citizens via EUDCC" could be made clearer as 1) Evidence Identification and Clarification; 2) Evidence-based Policymaking; 3) Policy Implementation for Citizen Benefit. The need for agility through timely, actionable information from science and field surveillance/monitoring of policy impacts could be emphasised more.

 The assertion that rapid antigen tests are less accurate than PCR tests is not true if the purpose is to identify people likely infectious across the whole infection course. PCR tests pick up infectiousness slightly earlier than antigen tests but go on to misclassify (as infectious) people who are no longer infectious. PCR is the gold standard test of having been infected but not the gold standard test of likely infectiousness. This is important if there is a societal cost to misclassifying non-infectious individuals as infectious, for example if it prevents a key worker maintaining critical infrastructure/services. See Mina, Michael J et al. (2021 [Ref 2]), which dealt with this misconception at the height of COVID-19 policy pressure (requiring rapid identification of likely infectious individuals). And see https://www.thepandemicinstitute.org/collaborate/academics/community-testing-review/ for reflections on clarifying evidence in this way across multiple testing purposes.

 The data and information systems focus of the article is useful. The authors might emphasise more the need to link digital workflows around certification. Burnside G et al. (2024 [Ref 8]) gives the example of a need to link test results with ticket (de)activation for entry to mass cultural events. Again, the purpose (for testing or certification) is key to optimising the policy. For example, the EU Digital Covid Certificate, is relevant to more than travel, and the wider uses of digital certificates may be important in the next pandemic.

 The authors might want to note how the EU Common List of COVID-19 Antigen Tests could have accumulated metadata from end-to-end testing evaluations - very few (e.g. Zhang X [2024 (Ref 3)] compare different manufacturer's devices after basic approvals - and there was very little information on novel uses (such as combinations) of devices in response to real-world pressures using readily available stocks. The authors might recommend surfacing natural experiments of pandemic testing.

Where applicable, are recommendations and next steps explained clearly for others to follow? (Please consider whether others in the research community would be able to implement guidelines or recommendations and/or constructively engage in the debate)

Partly

Does the article adequately reference differing views and opinions?

Partly

Are all factual statements correct, and are statements and arguments made adequately supported by citations?

Partly

Is the rationale for the Open Letter provided in sufficient detail? (Please consider whether existing challenges in the field are outlined clearly and whether the purpose of the letter is explained)

Partly

Is the Open Letter written in accessible language? (Please consider whether all subject-specific terms, concepts and abbreviations are explained)

Partly

Reviewer Expertise:

Public Health, Data Science, COVID-19/pandemic testing

I confirm that I have read this submission and believe that I have an appropriate level of expertise to confirm that it is of an acceptable scientific standard, however I have significant reservations, as outlined above.

## References

[1] : Put to the test: use of rapid testing technologies for covid-19. *BMJ* .2021;372: 10.1136/bmj.n208 n208 10.1136/bmj.n208 33536228

[2] : Clarifying the evidence on SARS-CoV-2 antigen rapid tests in public health responses to COVID-19. *Lancet* .2021;397(10283) : 10.1016/S0140-6736(21)00425-6 1425-1427 10.1016/S0140-6736(21)00425-6 33609444 PMC8049601

[3] : Can self-testing be enhanced to hasten safe return of healthcare workers in pandemics? Random order, open label trial using two manufacturers’ SARS-CoV-2 lateral flow devices concurrently and nested viral culture study. *BMC Infectious Diseases* .2024;24(1) : 10.1186/s12879-024-10155-z 10.1186/s12879-024-10155-z 39528957 PMC11555827

[4] : Performance of the Innova SARS-CoV-2 antigen rapid lateral flow test in the Liverpool asymptomatic testing pilot: population based cohort study. *BMJ* .2021;374: 10.1136/bmj.n1637 n1637 10.1136/bmj.n1637 34230058 PMC8259455

[5] : Evaluating social and spatial inequalities of large scale rapid lateral flow SARS-CoV-2 antigen testing in COVID-19 management: An observational study of Liverpool, UK (November 2020 to January 2021). *Lancet Reg Health Eur* .2021;6: 10.1016/j.lanepe.2021.100107 100107 10.1016/j.lanepe.2021.100107 34002172 PMC8114854

[6] : Recalibrating SARS-CoV-2 Antigen Rapid Lateral Flow Test Relative Sensitivity from Validation Studies to Absolute Sensitivity for Indicating Individuals Shedding Transmissible Virus. *Clin Epidemiol* .2021;13: 10.2147/CLEP.S311977 935-940 10.2147/CLEP.S311977 34703318 PMC8527245

[7] : Impact of community asymptomatic rapid antigen testing on covid-19 related hospital admissions: synthetic control study. *BMJ* .2022;379: 10.1136/bmj-2022-071374 e071374 10.1136/bmj-2022-071374 36418047 PMC9682337

[8] : COVID-19 risk mitigation in reopening mass cultural events: population-based observational study for the UK Events Research Programme in Liverpool City Region. *J R Soc Med* .2024;117(1) : 10.1177/01410768231182389 11-23 10.1177/01410768231182389 37351911 PMC10858718

# Response

**Published:** 2024-11-25

**Authors:** Mauro PETRILLO

*Dear Prof. Buchan,* *Thanks a lot for your valuable comments and suggestions that you have provided in the report.* *We will surely implement them, and wait for those of other reviewers, to provide a revised version of the manuscript.* *Best regards,* *Mauro Petrillo, on behalf of the authors.*

# Response

**Published:** 2024-12-12

**Authors:** Mauro PETRILLO

Important topic. Lessons from rapid antigen testing in COVID-19 responses that are key to future pandemic preparedness. The learning is also applicable to minimising avoidable gaps between evidence availability and policy implementation across a wide range of topics of public health importance.

***Author Response:*** *Thank you for your kind words and for recognising the importance of our topic. We are glad you agree that the lessons learned from rapid antigen testing in COVID-19 responses can inform future pandemic preparedness and have broader implications for public health policy. *  

 The article might better draw upon related multi-stakeholder reflections such as: https://www.thepandemicinstitute.org/collaborate/academics/community-testing-review/, where experts (mostly from the UK COVID-19 Testing Initiatives Evaluation Board) reflected on SARS-CoV-2 rapid antigen testing uses, evaluations and impacts.

***Author Response:*** *Thank you for bringing to our attention the valuable work and reflections of the Pandemic Institute and the UK COVID-19 Testing Initiatives Evaluation Board on SARS-CoV-2 rapid antigen testing. We appreciate the relevance of this work and acknowledge the importance of considering the broader perspectives of multiple stakeholders in the field. However, as our paper focuses on the integration of science into policy in the context of rapid antigen testing during the COVID-19 pandemic at European Commission level, and the lessons learnt in the process, a detailed comparison of these reflections is outside the scope of our current work. Nevertheless, we have taken the opportunity to mention your suggested reference in our paper in the “Actionable recommendations” section, as it can be useful for the readers of the paper. *  

 The article needs to make the purposes for testing (e.g. Ref 1, 9) clearer. Terms "effectiveness" "efficiency" "reliability" "accuracy" need a context of use and a defined utility e.g. for minimising the time taken to identify that a person is likely infectious and to inform their (or other person's) actions such as self-isolation, minimising social mixing, early return from quarantine or isolation to work.

***Author Response:*** *Thank you for your comment. We agree that clarifying the purposes for testing and providing context for the terms 'effectiveness', 'efficiency', 'reliability', and 'accuracy' is relevant. We revised different sections across the paper to better specify the importance of testing in specific scenarios, such as identifying infectious individuals and developing mitigation measures.*  

 The three stages of "information rationalisation", "integration of science into policy" and "benefits for citizens via EUDCC" could be made clearer as 1) Evidence Identification and Clarification; 2) Evidence-based Policymaking; 3) Policy Implementation for Citizen Benefit. The need for agility through timely, actionable information from science and field surveillance/monitoring of policy impacts could be emphasised more.

***Author Response:*** *Thank you for suggesting new titles for the three stages of the process. We agree that rephrasing them according to your suggestion provides a clearer and more concise structure. *  

 The assertion that rapid antigen tests are less accurate than PCR tests is not true if the purpose is to identify people likely infectious across the whole infection course. PCR tests pick up infectiousness slightly earlier than antigen tests but go on to misclassify (as infectious) people who are no longer infectious. PCR is the gold standard test of having been infected but not the gold standard test of likely infectiousness. This is important if there is a societal cost to misclassifying non-infectious individuals as infectious, for example if it prevents a key worker maintaining critical infrastructure/services. See Mina, Michael J et al. (2021 [Ref 2]), which dealt with this misconception at the height of COVID-19 policy pressure (requiring rapid identification of likely infectious individuals). And see https://www.thepandemicinstitute.org/collaborate/academics/community-testing-review/ for reflections on clarifying evidence in this way across multiple testing purposes.

***Author Response:*** *Thank you for pointing out the important distinction between PCR and antigen tests in identifying infectious individuals. We appreciate your clarification that PCR tests, while sensitive to early detection, can misclassify non-infectious individuals as infectious, whereas antigen tests may be more accurate in identifying those likely to be infectious across the course of the disease. We revised our text, across different sections, to reflect this nuance. We also appreciate the references you provided, including the work by Mina et al. (2021) and Crozier et al. (2021), which help inform our discussion on this topic.*  

 The data and information systems focus of the article is useful. The authors might emphasise more the need to link digital workflows around certification. Burnside G et al. (2024 [Ref 8]) gives the example of a need to link test results with ticket (de)activation for entry to mass cultural events. Again, the purpose (for testing or certification) is key to optimising the policy. For example, the EU Digital Covid Certificate, is relevant to more than travel, and the wider uses of digital certificates may be important in the next pandemic.  

***Author Response:*** *Thank you for highlighting the importance of optimising the use of certifications, particularly in high-risk settings such in social events, (as illustrated by Burnside et al. (2024). We implemented your suggestion in the Evidence Identification and Clarification, Policy Implementation for Citizen Benefit via the EUDCC and in the Conclusions chapters of the paper.*  

 The authors might want to note how the EU Common List of COVID-19 Antigen Tests could have accumulated metadata from end-to-end testing evaluations - very few (e.g. Zhang X [2024 (Ref 3)] compare different manufacturer's devices after basic approvals - and there was very little information on novel uses (such as combinations) of devices in response to real-world pressures using readily available stocks. The authors might recommend surfacing natural experiments of pandemic testing.

***Author Response:*** *We appreciate your suggestion to view the EU Common List of COVID-19 Antigen Tests as a potential source of metadata. You raise a crucial point on the lack of comparative studies between different manufacturers' devices beyond basic approvals, as well as limited information on innovative uses of devices in response to real-world challenges. We addressed this comment at the end of the chapter “Evidence Identification and Clarification”.*
